# The Potential for Conservation Tillage Adoption in the San Joaquin Valley, California: A Qualitative Study of Farmer Perspectives and Opportunities for Extension

**DOI:** 10.1371/journal.pone.0167612

**Published:** 2016-12-01

**Authors:** Anne V. Bossange, Kandace M. Knudson, Anil Shrestha, Ronald Harben, Jeffrey P. Mitchell

**Affiliations:** 1 Department of Land, Air and Water Resources Department, University of California, Davis, California, United States of America; 2 John Muir Institute of the Environment, University of California, Davis, California, United States of America; 3 Plant Science Department, California State University, Fresno, California, United States of America; 4 California Association of Resource Conservation Districts, Arroyo Grande, California, United States of America; 5 Department of Plant Sciences, University of California, Davis, California, United States of America; Agroecological Institute, CHINA

## Abstract

Conservation tillage (CT) systems have a number of potential benefits including lower crop production costs and the ability to reduce soil erosion that have made them common in several regions of the world. Although CT systems have been researched and successfully implemented on some farms in California’s San Joaquin Valley (SJV), overall adoption is low and the reasons for the region’s comparatively low rates of adoption are not known. In 2011, we conducted written surveys and interviews with SJV farmers to identify characteristics of farmers who adopt or do not adopt CT, to determine reasons for non-adoption of CT, and to learn how successful CT adoption takes place in the SJV. We found that a universally acceptable definition of CT needs to be developed in order for effective research, outreach and communication on CT. Our research, which examined CT adoption within the expected progression of the diffusion of innovation model, suggested that larger and less diverse farms were more likely to use CT. Most farmers expressed transition to CT as a continuous learning process. Further, we conclude that gaining meaningful experience with CT practices by researchers in the local context is also a large component of successful adoption.

## Introduction

Conservation tillage (CT) has become commonplace in several regions of the world as a solution to erosion problems [1, 2] and a strategy for reducing production costs [3, 4, 5]. In addition to reducing erosion, CT has also been shown to increase surface soil organic carbon (C) [6], increase infiltration and water-holding capacity [7], and lower soil temperatures [8]. Use of CT practices has been responsible for a renaissance of farming in several diverse regions of the world including the US Great Plains [9], the central Canadian plains [10, 11], much of Brazil, Argentina and Paraguay [12, 13], and Western Australia [14, 15]. Both the recorded histories of the development of CT systems now used in various regions of the world [16] and studies of the specific economic and environmental drivers that motivated their adoption in these regions are fascinating and complex. The local technologies, people and social networks that led to transformations toward CT across these regions have been archived in a variety of accounts [10, 16, 17] and formats [18]. Understanding how improved tillage systems are adopted is important because it sheds light on how transformational changes are made in agricultural production systems [10, 11].

**Fig 1:**
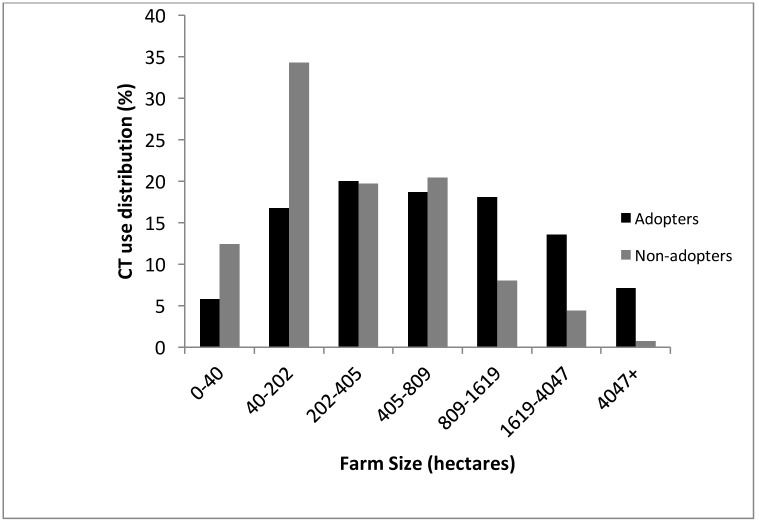
Farm Size of CT Adopters and non-adopters.

Over 350 different crops are grown in California and it has been the highest producing US state over the past 50 years [19]. The San Joaquin Valley (SJV) in California (Fig 1) is a remarkably productive agricultural region in the southern part of the State’s Great Central Valley [20]. Mounting evidence from the region’s persistent recent drought [21] and the likelihood that water, energy, and labor constraints are likely to intensify in the future have recently led to the suggestion that reduced disturbance CT systems that have become prominent in these other regions may also have relevance for the SJV [22]. Although CT systems have been researched and successfully implemented on some farms in California’s SJV, overall adoption in the region remains low [23, 24]. While water erosion is not perceived as a problem on farms in the SJV due to the region having a generally flat topography and because land leveling is commonly done for irrigation, CT can play a role in long-term soil quality improvement [25], increased water use efficiency [26], reductions of overall farm implement passes, and the need for labor and production costs [27]. Drivers or motivating factors for the adoption of CT in the SJV are likely to be different from those that have been responsible for the much higher adoption rates in other regions of the world [24, 28].

Research on the technical aspects of cropping systems under CT in California has been conducted since 1994 by a group of farmers, researchers, government, and private sector partners who make up the Conservation Agriculture Systems Innovation (CASI) workgroup [24, 28]. Recent publications document and detail the impacts of CT practices in the SJV on crop yields and production costs [27, 29, 30], and also soil [30] and air quality [31] improvements with CT. A 2007 study by Mitchell et al. [28] reported very little adoption of CT by farmers in the region, with the top reasons for non-adoption including ‘lack of information,’ ‘belief that CT won’t work with current rotations,’ and farmers being generally ‘content with current practices’. That study is the only study to date of farmer attitudes toward CT adoption in the SJV and it gave rise to the more detailed behavioral change research reported here. It is important to point out that because the use of CT practices has been considerably more challenging and therefore far less widespread in the generally high-valued annual cropping systems of regions such as the SJV and the Low Desert regions of California and Arizona, or in the largely vegetable production systems of Texas or Florida, investigations on the adoption dynamics for CT in intensive production regions such as these are lacking.

Qualitative research about the social aspects of agricultural innovation is increasingly recognized as a necessary component for understanding the behavioral complexities that influence the widespread adoption (or diffusion) of innovation [32]. Work shows that successful adoption of innovative conservation practices will be enhanced if awareness of the concerns, ideas, and varying perspectives of the target community are included in the design of extension processes [33, 34]. Research on adoption of innovative agriculture practices in other regions has found social aspects to be very significant to the adoption process [35, 36, 37, 38, 39]. Studies investigating adoption of CT in different regions have reported that variation in each place led to different CT adoption narratives and processes [16]. Furthermore, adoption of CT in the Midwest U.S. and Australia required a paradigm shift from plow culture to minimal disturbance [16]. Since soil tillage is a major management tool to prepare seedbeds, kill weeds, and incorporate fertilizers, adoption of CT required ‘significant managerial skill’ to relearn how to manage each cropping challenge that tillage had previously addressed [35].

In Australia, Brazil, and the Midwestern U.S., soil loss due to wind and water erosion has been a significant contributing factor in the development of policies for CT adoption [40, 41, 42]. The study by Mitchell *et al*. (2007) [28], however, reported that the impetus for farmers to adopt CT in the SJV were fuel and labor cost savings from tillage pass reduction and water savings from residues preserved on the soil surface rather than prevention of soil erosion. A meta-analysis of adoption of numerous different best management practices (BMPs) in the U.S. found that ‘education levels, capital, income, farm size, access to information, positive environmental attitudes, environmental awareness, and utilization of social networks emerge as some of the variables that are more often positively associated with adoption rates’ [43]. For example, in an Iowa- based CT adoption study, demographics such as age, education, farm size, and gross income differed significantly between adopters and non-adopters [35]. In addition to demographics, farmer perceptions of the risk, and the extent of the environmental problem, and their assumptions about local acceptance and adoption of CT also correlated with adoption and non-adoption [35]. Another Iowa minimum tillage study found that adopters have a lower average age and larger average acres owned [44], while in an Ohio study, it was reported that larger farms were less likely to use soil conservation practices [45]. It is important to note that farmer characterizations to explain adoption of CT have been inconsistent across locations and studies [46].

Adoption studies can be used to inform policy and extension support and delivery. It has been recognized that farmers are more likely to adopt innovations that are less complex [34, 47]. Conservation tillage is an innovation that requires extensive change and, as Coughenour and Chamala, (2007) [16] describe, new tillage systems are not designed by researchers or industry and then diffused to farmers, but rather they are developed by farmers and each location and cropping system requires different context-based innovation. Study on adoption of innovation has moved away from the diffusion model of Rogers (1961) [48] toward an understanding that complex agricultural innovation is a process that cannot be easily predicted or planned [49] and is based on many variables [50]. Innovation includes the process of communication and facilitation of ideas as well as the context and interpretations that are being made by involved members. Thus, in extension work involving adoption of innovation, understanding the social, economic, and political context is central to success [51].

Since innovation processes and adoption outcomes are place-based and context-specific [35], each agricultural community needs to understand the local reasons for adoption and non-adoption of desired practices. Therefore, there is a need to examine some of the variables responsible for slow adoption of CT in an intensive production region such as California’s SJV. Survey and interview approaches need to combine both demographics and responses to attitudinal changes as quantitative indicators of variables that explain both adoption and non-adoption and to obtain an in-depth qualitative understanding of the farmer perspectives on CT. This understanding of where, why, and how CT innovation has been happening in the SJV can give insight into specific cropping systems or farmer groups that are more likely to adopt if given resources and support are available and the information could help local extension professionals design programming that supports this process. In addition, the information may also be of use to other areas where intensive production systems are prevalent. Therefore, the objectives of this qualitative study were to: i) identify characteristics of farmers who adopt or do not adopt CT, ii) determine the reasons for non-adoption of CT in the SJV, and iii) learn directly from farmers what it takes to successfully transition to CT in California cropping systems.

## Materials and Methods

This study used the standard behavior change social science research tools of surveys, interviews, and notes and observations from facilitated meetings with farmers. These techniques provided a conceptual framework to examine the process of CT adoption in this intensive production region. Interviews were designed to reveal farmer reasoning behind the data that were collected and analyzed in the survey. The inductive nature of this qualitative research allows the answers to emerge and isn’t meant to “test” deductively constructed assertions or hypotheses about behavior.

### Survey

In 2011, a survey was mailed to 2500 tomato, cotton, and dairy silage farmers as well as other agriculture professionals across the SJV using mailing lists provided by the California Tomato Research Institute, the California Cotton Ginners and Growers Association, and the Western United Dairymen commodity grower groups. Members of these commodity groups constitute the overwhelming majority of farmers of these crops and therefore represented a very broad cross-section of SJV farmers for these historically important, major annual crops [19]. The survey consisted of questions such as, beliefs/attitudes and reasons for adopting CT, farm/farmer demographics (age, experience, education, farm size, crops grown), information sources, and one open ended question, ‘Please tell us in your own words why you do or do not use conservation tillage.’ The definition of CT was given at the beginning of the survey as ‘Conservation tillage involves no-till, strip-till, or minimum tillage systems that reduce overall tillage passes by at least forty percent relative to your region’s conventional tillage practices.’ Because of the very wide range of actual tillage systems and descriptions that are commonly used in California [23, 24], we believed that it would be important to identify both the classic forms and terms of CT used generally in other regions (no-tillage, strip-tillage), and also the broader ‘minimum tillage’ category that we have formally identified and introduced in California in previous publications. Both the survey and the interviews (see below) were conducted in compliance with the Institutional Review Board at the University of California, Davis under IRB #215143.

### Survey data analysis

Data from the survey were analyzed using one-way ANOVA [52] to determine differences between adopters and non-adopters. Results were confirmed using parametric and non-parametric analysis (chi-square and Wilcoxon). Significance was determined when p < 0.05. Demographics and beliefs and attitudes including age, number or years farming, farm size, and education, were tested for correlation to adoption. Crops grown were compared to adoption of CT and to farm demographics. The number of listed crops grown by each farmer was totaled and compared to CT adoption and farm size. Additionally, crops listed in ‘other’ were tallied to get a sense of crops that farmers were growing that were not included in the survey.

### Interviews

Semi-structured interviews were conducted with seven farmers to better understand SJV farmer perspectives. The interviewees were selected from a list of farmers who had some contact with the CASI workgroup, thus all farmers had some exposure to the CT work being done by this group. These interviewees (sample) represented CT adopters, non-adopters, and two who had tried elements of CT. The interviewees characterized as adopters practiced forms of CT that would meet the definitions of CT in regions where residue preservation is included in the definition [53]. The interviewees classified as adopters had over 40% pass reduction and over 30% residue preservation. All the interviews were conducted and voice-recorded on or near the participant’s farm. Interviews lasted around one hour and were transcribed in Express Scribe [54] and analyzed in Atlas.ti 6.2 [55]. Participants provided verbal informed consent to participate. This consent was sufficient to them and was in compliance with the Institutional Review Board at the University of California, Davis under IRB #215143. The interviews were analyzed using open-coded constant comparative analysis [56]. Using this method, each sentence was assigned a code(s) that represented the idea or meaning being expressed. The initial coding revealed emergent themes across transcripts and a second analysis was used to refine the codes. Emergent themes were then compiled and a deeper coding of dominant themes was performed. Additionally, a list of barriers was generated and all interviews coded based on references to these barriers. Findings from the interviews were based in the words of farmers and all farmers at one point or another referenced the importance of context before giving a response.

### Additional field notes

Over the two years of the study, many CASI workgroup events and personal interactions also informed this work and analysis. These events included participation in field days, CASI meetings, and farm visits in addition to conversations with CASI members, CT researchers, and participants. Field notes were taken at the West Side Research and Extension Center presentation of the 2012 Conservation Tillage and Controlled Traffic Farming Conference and at various CASI field days and presentations. These events contributed to the overall understanding of the on-going work promoting CT in the SJV. Interpretation of results thus took into account information from the survey, interviews, and field note data by cross checking themes from each component with evidence from the others.

## Results and Discussion

### California context as shared by farmer interviewees

All farmers communicated the complexity of their individual farms. They discussed the high number of crops in many cropping systems as a major difference between CT in the SJV and areas of the country that have higher levels of CT adoption. A vegetable farmer described how the diverse mix of crops he grows makes it difficult to use CT, ‘Our crop mix just doesn’t lend itself to conservation tillage…we change bed configuration possibly every year’. Farmers also pointed out that, when comparing the adoption of CT in the SJV to other parts of the country and world, it is important to acknowledge that lessons learned in other regions may not transfer to the SJV. One farmer expressed that, ‘With land values here and farming costs here, our rotations are higher value rotations. I think where the major success in conservation tillage is coming about is in the Plains areas, rainfed, low input areas, where those types of operations apply or lend themselves better to conservation programs. We're so high cost, high value you could not grow corn and soybeans only in California and stay in business whereas in the Midwest they can do that and stay in business. I mean you just couldn’t do that here you couldn’t have that rotation here because of our growing costs.’

Another context given by all farmers was the importance they place on an economic justification before making the transition to any new practice. One farmer described the necessity of considering the economics, ‘But at the end of the day it still has to work economically… I mean it’s better staying in business than out of business’. Many of the farmers made sure to stress this point and to clarify that even conservation based practices need to work out economically.

### Survey responses

A total of 402 surveys were returned for a participation rate of 24% (returned by 307 farmers and 95 non-farmers). The non-farmer responses were recorded but not used in the data analysis presented. The low survey response rate (24%) could be due to the mail mechanism of distribution to tomato, cotton, and silage commodity mailing lists. These are broad lists with uncertainty about the number of actual farmers with decision-making responsibilities that received the surveys. Of the 307 farmer surveys, 161 (53%) of the farmers reported that they had adopted CT (Table 1). Of the farmers reporting non-adoption of CT, 36 (25%) reported that they have tried CT in the past. To the question ‘How much do you know about CT?’, on a scale of 1 to 7 (where 1 = nothing and 7 = a lot), the average knowledge of the farmers who used CT was significantly higher (4.97 vs 4.05) than those who did not use CT (Table 1).

**Table 1:** Farmer demographics for non-adopters and adopters show difference in mean farm size and little difference between age, education and experience farming. For questions where responses were reported as a range, means were calculated using the middle of the range.

	Non-adopters	Adopters
	n = 142 (47%)	N = 163 (53%)
**Age**		
**30–40**	14	11
**40–50**	21	34
**50–60**	60	63
**60–70**	32	37
**70–80**	9	8
**80–90**	1	1
**Mean**	55.3	55
**Farm Size (n) Hectares**		
**0–40**	17	9
**40–202**	50	28
**202–405**	28	33
**405–809**	28	30
**809–1619**	12	30
**1619–4047**	6	22
**4047+**	1	11
**Mean Farm Size**	1175	2654
**Mean number of years farming**	26.7	27.4
**Education completed**		
**High School**	36	39
**College**	88	106
**Graduate**	14	18
**Other**	3	0

### Interview data emergent themes

The qualitative data analysis revealed a number of emergent themes. The top themes are listed in Table 2.

**Table 2:** Farmer ‘beliefs’ about conservation tillage. Farmers were asked to ‘please respond to the following statements based on what you ‘believe’ about conservation tillage… ‘1’ is strongly disagree and ‘7’ is strongly agree.’ Responses for adopters of CT and non-adopters of CT were ranked with gray boxes representing the top ranked beliefs.

		CT adopters			CT non-ad			
#	Questions	Mean	Rank	SD	Mean	Rank	SD	Signif.
**1**	How much do you know about conservation tillage?	5.01	na	1.14	4.05	na	1.41	*
**2**	I am satisfied with my current practices.	4.53	1	1.74	5.62	1	1.33	*
**3**	My production practices are already about as ‘lean’ as they can be.	4.33	2	1.62	4.99	3	1.54	*
**4**	Conservation tillage is being promoted by environmentalists	3.98	3	1.81	4.50	9	1.82	*
**5**	Converting to conservation tillage requires too much new equipment	3.71	4	1.54	4.67	7	1.50	*
**6**	I really see little reason to change what I'm currently doing.	3.61	5	1.73	4.82	5	1.73	*
**7**	The ‘benefit/risk’ case for conservation tillage in California has not been proven to me.	3.47	6	1.80	5.22	2	1.43	*
**8**	There is not enough demonstrated and successful experience with conservation tillage in California.	3.43	7	1.73	4.43	13	1.65	*
**9**	Conservation tillage fields will not yield as well as conventional tillage fields.	3.36	8	1.64	4.85	4	1.45	*
**10**	Conservation tillage is really not suited to California's very diverse crops.	3.34	9	1.60	4.44	12	1.44	*
**11**	Conservation tillage results in more crop disease and weed-related losses.	3.31	10	1.69	4.39	14	1.40	*
**12**	The costs of converting to conservation tillage are too high.	3.30	11	1.46	4.48	11	1.46	*
**13**	There is not enough public information available about conservation tillage in California.	3.30	12	1.70	4.12	17	1.65	*
**14**	There is not enough technical information and support available for conservation tillage in California.	3.16	13	1.47	4.00	19	1.63	*
**15**	Conservation tillage practices will likely result in unacceptable yield losses.	3.14	14	1.62	4.66	8	1.54	*
**16**	Conservation tillage requires too many changes in what I'm currently doing	3.09	15	1.35	4.79	6	1.44	*
**17**	Conservation tillage costs too much even with USDA cost sharing.	3.00	16	1.45	4.25	15	1.42	*
**18**	Conservation tillage requires too much equipment ‘know how’ and attention.	2.90	17	1.33	3.76	21	1.43	*
**19**	Conservation tillage will not work for my crops.	2.86	18	1.57	4.50	10	1.63	*
**20**	Conservation tillage will not work in California soils where there are no freeze-thaw conditions like in the Midwest.	2.78	19	1.50	4.08	18	1.52	*
**21**	Conservation tillage is too risky.	2.73	20	1.27	3.98	20	1.24	*
**22**	I don’t have time to deal with learning about and securing new equipment for conservation tillage.	2.71	21	1.50	3.63	22	1.46	*
**23**	Conservation tillage won’t work with my irrigation systems.	2.68	22	1.56	4.21	16	1.59	*
**24**	I don’t know enough about conservation tillage equipment so that I can work on it if breaks.	2.43	23	1.39	3.31	23	1.50	*

* = 0.05 level of significance.

### Characterization of CT adopters and non-adopters

The age of the farmer and the number of years they were farming were similar between the CT adopters and non-adopters. Additionally, education level did not vary either between the two groups (Table 1). The average age of farmers who participated in the survey was 51 and the average number of years farming was 27. However, the average farm size of the CT adopter group was greater (1074 ha vs 476 ha) than the non-adopter group (Fig 1).

Interviews with farmers who had adopted CT revealed that more highly trained labor hours were necessary in the transition to CT due to the regularity of new management challenges. One farmer described the time that he had to invest to learn to use CT as ‘The challenge was to make time to go out there… the challenge was to walk the field every day’. Increased effort and commitment were described by all farmers practicing CT.

**Fig 2:**
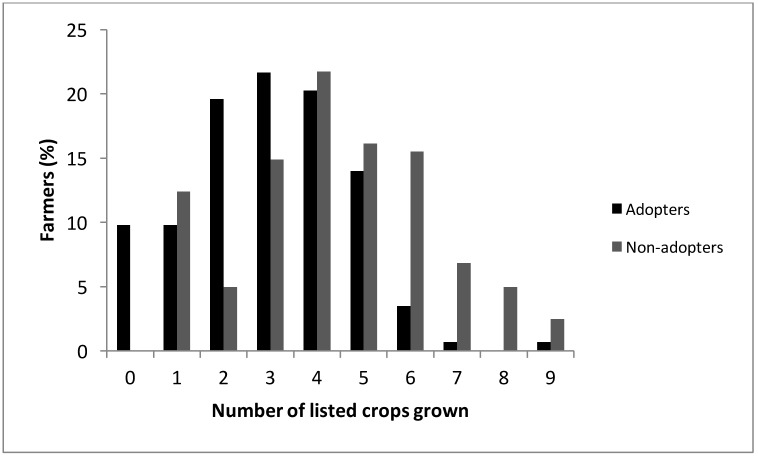
Crop diversity of CT adopters and non-adopters as shown by the number of crops grown.

**Fig 3:**
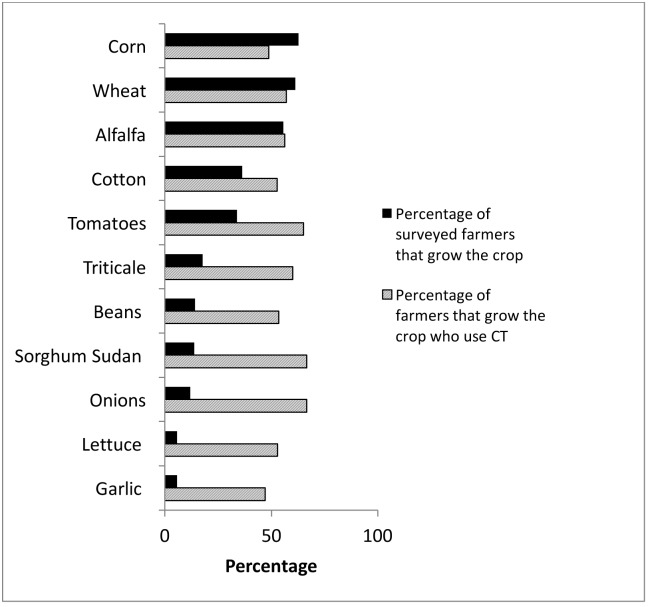
Crops grown and percentage of survey respondent farmers using CT for each crop.

CT adopters grow fewer crops than non-adopters. The distribution of total crops grown for each group is shown in Fig 2. An additional note is that there were 14 CT adopters who did not grow any of the listed crops and 10 of those farmers mentioned that they grew tree crops or grapes. The most consistently grown crops (of those listed in the survey) and the percentage of farmers that grow each crop who use CT are presented in Fig 3. When farmers indicated that they grew multiple crops, the survey did not distinguish which of those crops are farmed with CT. The percentage of CT adopters who grew tomatoes was higher than the number of non-adopters growing tomatoes. However, CT adopters and non-adopters were equally likely to grow the other crops listed (alfalfa, beans, biofuel, corn, cotton, garlic, lettuce, onions, sorghum sudan, triticale, and wheat).

### Farmer perceptions about aspects of CT

Responses to questions about CT, presented in table 2, reveal farmer attitudes toward different aspects of CT practices. The series of beliefs are ordered by level of agreement for each farmer group, adopters, and non-adopters with higher mean values associated with higher agreement with a given statement. Both groups (CT adopters and non-adopters) agreed most strongly with ‘I am satisfied with my current practices’. While the means for each question were significantly different for each group, adopters and non-adopters shared three of their five top ranked beliefs (Table 2, in dark gray). The responses outlined in black (Table 2) represent those that are the strongest predictors of adopters and non-adopters of CT using a stepwise model in JMP (the model accounted for 38% variation). Non-adopters identified all statements as larger barriers to adoption than adopters (Table 2).

### Barriers to adoption of CT

Adopters of CT consistently had different responses to the questions in Table 2 than the non-adopters. These differences could correlate to differences in personality types among people who switch to CT or it could be a result of a shift in perspective on the practices as a result of converting to CT. The process of converting to CT likely results in a significant knowledge gain by farmers and success with the practice could lead to a more favorable outlook. Farmers currently using CT have accumulated experience-based knowledge of CT resulting in a clearer idea of whether or not these barriers were true for their operation. It can be expected that farmers who successfully practice CT will have a far more favorable attitude toward the practice. If they are not successful in adopting CT, then they will have reinforced their previous perception of CT: that it won’t work on their farm. Additionally, farmers practicing CT have already overcome barriers to adoption and may no longer perceive the challenges that they faced as barriers.

### CT doesn’t work in our system

This code encompassed a range of comments that basically said, ‘I can’t use CT because [any number of variables that farmers cite here].’ The reasons given include: diversity and type of crops grown, irrigation system incompatibility, regulations for control of certain pests, and soil type (Table 3). Unlike many agricultural regions where relatively few crops are grown, the diversity of SJV crops (200–400 crops grown in the SJV) means that farmers are going to have very different farming experiences depending on what they grow and marketing requirements for each crop [41].

**Table 3:** Interview emergent themes. The recurring coding themes listed in the table represent broad sentiments expressed by interviewed farmers. Quotes and elaboration are reported in the text.

Interview coding themes
Top Emergent Themes:
• ‘Economics is an important factor in CT adoption’
• ‘CT doesn’t work in our system’
• ‘CT requires a change of mindset’
• ‘CT requires a lot of learning’
• ‘Good local examples of CT are required for adoption to happen’
Technical issues with CT:
• Irrigation incompatibilities with CT
• Crop diversity and many specific vegetable crops are not compatible with CT
• Equipment issues are a barrier to CT adoption

In the comments and interviews, some farmers attributed their attitude that ‘CT won’t work in our system’ to hearing about or seeing other farmer’s fields in transition and not liking what they heard or saw; in other words, they were making a subjective evaluation of CT. One farmer discussed how detrimental a visible trial failure can be if neighbors saw one failure and assumed that the practice would never work. This farmer mentioned that when he was learning about something new, he conducted his trials away from any roads so that if he ran into challenges or made mistakes others did not write off the whole endeavor. This highlights the possibility that farmers may decide whether a system works or not without having experience of their own on the practice.

It was evident that some systems do lend themselves more easily to CT than others. In interviews, farmers shared that CT or reduced passes were more common in certain crops in California including tomatoes, forage, cereal crops, and perennial tree systems. On the other hand, cropping systems with diverse vegetables with changing planting bed configurations were more difficult systems for CT use because of the need for tillage to reform beds and create uniform seed beds.

### Economics

**Fig 4:**
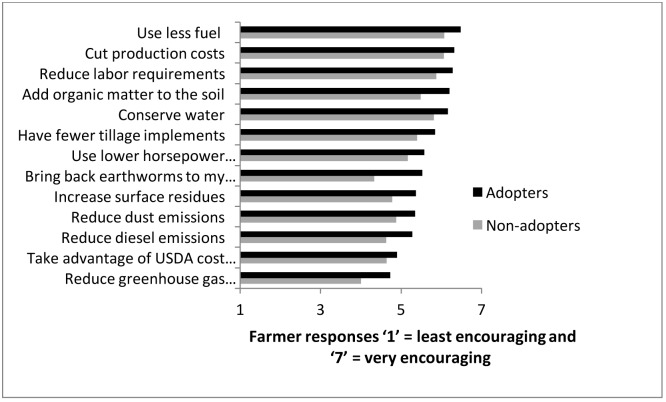
Ranking of reasons that encourage CT use by adopters and non-adopters.

Farmers generally expressed that they were interested in seeing economic benefits for practices in order to adopt them. A highly diversified grower described his economic perspective in the following words, ‘We’re under a lot of pressures, lack of water, financially we have to yield, fuel is very expensive’. Fig 4 demonstrates that the three most common reasons for adopting CT are directly related to cost savings. Some farmers opined that the economics of the crops that they grew and their familiarity with the common cropping systems in the SJV are barriers to adopting CT. Yet, farmers who had adopted CT said that they were able to maintain the same profit margin or more. Other farmers assumed that CT meant a loss of flexibility and crop yield and were concerned that they would not be able to adapt as easily or quickly to a changing economic climate if they committed to a CT system.

To be able to adopt CT, farmers felt the need for reliable economic analysis showing the monetary benefits before transitioning to CT. One farmer mentioned that farmers who had cheap water in their areas had little reason to invest in water saving practices such as drip irrigation because the savings were not comparable to areas that had expensive water. It was farmers with more expensive water who were more rapidly changing over to water efficient systems. Other farmers mentioned that many SJV farmers were doing well economically using conventional cropping systems and did not feel the economic pressure to change. On the question on why people were not transitioning to CT, one farmer mentioned, ‘I think a lot of them have had success with the conventional way of doing it and I just think for them it’s a risk…so I just think they're playing it safe. Hey I’ve done it this way, I do [well enough], so there’s no need for me to try and change.’

### Why do farmers adopt and what does it take?

The three main reasons for adopting CT were, reduction of fuel, production costs, and labor requirements (Fig 4). While the adopters and non-adopters of CT had significantly different responses for each question, the two groups ranked the three reasons in a similar order. Therefore, short-term economic profit seemed to be the overarching reason for adoption or non-adoption of CT (Fig 4). Environmental and conservation reasons were not among the top three responses. Interviews also supported survey findings that economics was a strong reason for the use of CT. In the words of one farmer, ‘[CT] probably helped keep us in business because labor costs, fuel costs, seed costs, and everything else had gone through the roof. That was the one place we were able to save a lot of money and make better crops.’

### Information

**Fig 5:**
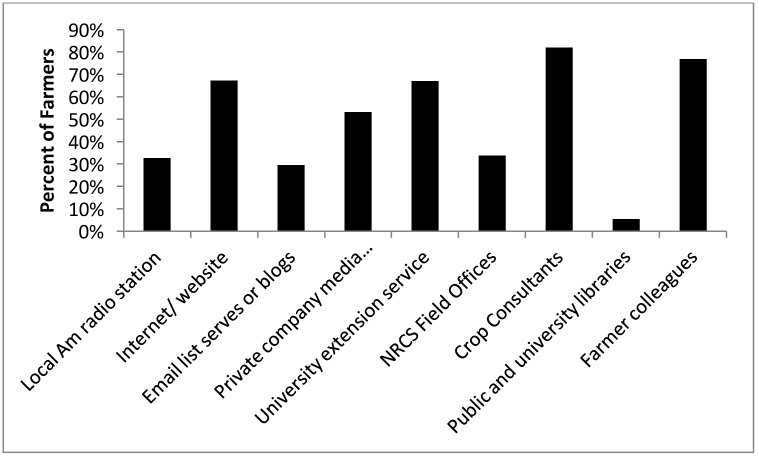
Percentage of farmers who indicated each information source was in their top five most used sources.

Results showed that, the top-most trusted source of information for farmers was crop consultants followed by farmer colleagues, university extension service, internet/website, private company media (pamphlets, and brochures), USDA Natural Resource Conservation Service (NRCS) field office personnel, email list serves or blogs, local AM radio station, and public university libraries in a highest to lowest ranking basis. The ranking of these information sources was similar between the adopters and non-adopters of CT (Fig 5).

### CT requires a full change of mindset

CT adopters described that they had made a big shift in their farming approach when they transitioned to CT. Furthermore, they mentioned that it was not just the shift in technical aspects, but they had to spend a lot of time out in the fields working to understand the new system. They added that they had to be involved in parts of the operation that normally fell under the responsibility of their employees. Farmers had to spend a lot of time in the field observing and getting opinions and advice from different people. In the words of one farmer, ‘Now the mindset had to change because our irrigation water was running faster since we hadn’t broke the soil down so much.’ Those who had been using CT described strong belief in the practices. They discussed in detail on how the soil had changed, or how they made decisions based on the way that water moved in their field, or how much carbon their soil needed. Another farmer quoted that, ‘In about five years the soil went from being ROCK hard to being pliable to where I could start to plant things. When we first started no-tilling, my big problem with conservation [tillage] or our perceived problem was going to be what are going to do with all that residue? It’s going to start accumulating here and how are you going to be able to plant through all that stuff? What we quickly learned was, where is all of our residue? It’s gone. The sun was oxidizing it. The worms were eating it up. So then I had to learn to start to grow different crops just to get some residue on the soil. For example I grow wheat not because I want to grow wheat but because I can get some residue that will last longer than eight months.’

On the other hand, many farmers did not seem to have the mindset that they needed to make a complete change. For example, on farmer stated that ‘For right now I just feel like we're at a happy medium where we're not totally conventional, [and] we're not totally no till, but it’s working.’ The responses of CT adopters suggested that farmers would need to break out of their current, conventional way of thinking about farming. Examples that farmers gave about the mindset change that is required to be able to adopt CT included: going against the way their family farmed or was farming; necessity to free up time and/or resources to understand whole system changes and be ready to sustain this additional learning time for a number of years during their transition to CT; and lack of a serious and compelling factor for adoption of CT, such as soil erosion in many parts of the world.

### CT requires a lot of learning

CT adopters described the process of problem-solving as requiring a lot of learning during the transition to CT. These included the management issues related to residue, salt accumulation with subsurface drip irrigation, and the timing of operations. During the transition to CT, the ecological aspects of the system continued to change and so did the learning process. Thus, farmers not only had new variables to consider during the transition, but year after year, they had to observe changes and adapt techniques accordingly. Again, in the words of one farmer, ‘… we haven’t figured out yet how to maintain that; when we rotate into tomatoes, because you have to pull beds and it’s all drip [irrigated] -sometimes after one year, two years, or three years—you have to pull the [buried drip irrigation] tape out just to rotate into safflower or wheat crop in order to reclaim that soil because there’s too much salinity on the surface of the soil from the drip tape water quality and we haven’t figured out that transition.’ This continuous ‘figuring out’ was referred to in multiple interviews and it can be concluded that, for SJV farmers, transition to CT was a continuous learning and problem-solving process.

### The technical issues

The results reflected that the various technical or system issues that farmers cited were based on their experience of CT being a continuous learning process. The specifics of the issues were different depending on the physical environment of the farm (e.g., soil, water source), the business structure of the farm (e.g., crops grown, farm size, access to capital), and the perspective of the farmer (e.g., satisfied or not with practices, environmental concern, social acceptance, and aversion to risk). Therefore, these technical issues need to be solved for more widespread adoption of CT. A recently-initiated private sector rental program for strip-tillage and precision planting equipment for silage corn is a highly successful example that provides technical support with state-of-the-art implements as a means for prospective CT farmers to learn and to become familiar with CT prior to purchasing new equipment.

### Definition of CT

One unexpected, yet potentially the most important finding of this study, was the definition of CT in California. The definition of CT as stated earlier in the description of the survey veered from other regions in the exclusion of a residue preservation requirement [53] and this reinforces the recent urging of Reicosky [24, 57] for more standardized terminology related to tillage system specifics.

Originally the definition of CT in California was made very broad because CT as practiced in other regions seemed unobtainable in California cropping systems because of residue issues [6]. A less restrictive definition that did not include the residue preservation requirement was chosen because farmers would be more likely to attempt reduced tillage systems than residue preservation systems [6]. While the strategy proved effective at being an inclusive definition, as evidenced by 50% of farmers reporting current CT use (Table 1), it is important to determine whether the practices these farmers are using contribute to improved soil health, one of the major benefits of using CT. The lack of clarity on the definition of CT can also be an issue in the interpretation of the results. An NRCS report from 2004 reported less than 1% adoption of CT in California [43]. The NRCS used the ‘residue preservation’ definition of CT. However, in this present study, 50% of survey participants declared use of CT based on the definition provided in the survey (Table 1). Therefore, the results of this survey are strictly based on the California definition of CT as provided in the survey and not on the NRCS definition. Nevertheless, a number of factors contributing to this report on the adoption of CT, include reduction in tillage passes and shallower tillage in the past ten years with the installation of buried drip [58], improved equipment and technology, and the perception among farmers that they are now achieving reductions of 40% or more in tillage passes [23]. In a 2002 survey of 290 SJV farmers, 50% had prior experience with CT and two-thirds of these were with minimum tillage practices [28]. While this is a step towards reducing overall soil disturbance, these reduced tillage practices generally involve considerable soil disturbance between each crop and do not maintain residues.

Interviews also confirmed confusion about the term ‘conservation tillage’. Before starting interviews, a number of farmers asked which definition of CT we would be using. For the purpose of this analysis, we used the self-reported categories from the survey to describe adopters and non-adopters with awareness that this is a broad definition that likely encompasses both farmers using no-till practices and farmers using the more general pass reduction or minimum tillage practices experienced in the SJV.

## Discussion

### Definition

Reported CT rates in the whole US are expected to be higher than actual due to different perceptions of the definition [59] corresponding to California survey results. The confusion around the definition of CT in education and extension efforts could lead to a disconnect between the practices and the reported benefits [59]. While California has unique aspects of its agricultural practices that led to a divergent definition, CT information from agricultural systems all over the world is used by the farming and research community. A definition consistent with literature on CT used elsewhere could reduce confusion around the terms. In the CT research community spanning many regions, there is a conversation about standardizing the definitions of reduced tillage practices in general due to confusion when interpreting research data coming from variations on these practices [60]. The Derpsch work emphasizes the need for reporting specifics about the methods and technologies used and standardization of research design to avoid very divergent research results and misunderstanding of these systems. Clarity of communication can lead to more relevant learning about these systems from the local to the international level.

### Characterization of adopters and non-adopters of CT in California

Prokopy et al. (2008) [43] and Bultena and Hoiberg (1983) [35] reported that education levels, farm size, and age, are correlated with Best Management Practice (BMP) and CT adoption. However, in our study farm size and number of crops grown were the only measured factors that were correlated with CT adoption in California. California has a highly educated farmer population with the majority of farmers surveyed having completed college level education or higher (Table 1) and this could have contributed to the difference in findings with abovementioned studies.

The positive correlation between farm size and CT adoption could relate to available capital to buy equipment and to take risk. On larger-scale farms, use of land and employee hours to run trials on unfamiliar operations may have less impact on the overall operation, which may be important considering the ‘learning-related costs’ that Lewellyn (2007) [61] cites as one contributor to slow adoption. If a farmer only farms one or two crops, they can concentrate their time into determining how to make CT work and get a clear sense for how reduced passes, labor, and fuel use all fit together and impact overall economics of the farm. On a farm with many crops, determining the details of using CT on a large diversity of crops while adhering to the principles of CT becomes much more complex and it could be more difficult to have a clear sense of how these practices impact the overall economics of the farm. In general, both adopters and non-adopters of CT reiterated that economic justification is important in adoption of a new practice which is consistent with the findings in other regions. In order for farmers to have confidence in the economics of CT, access to information on the technology required and the associated risks during the transition are valued by farmers [3].

The relationship between tomato growing and CT could be a result of the importance of tomatoes as a cash crop in California. Larger farms are likely to have some acreage of tomatoes in their operation as it is a very profitable crop in Central Valley. Another possibility is that many tomato growers may see themselves as CT practitioners due to the use of subsurface drip irrigation systems that do not require as much tillage as furrow irrigation systems. With the introduction of subsurface drip, most farmers have been able to dramatically reduce the number of passes during the season due to not having to land level and furrow the ground for flood irrigation. For air quality improvement, NRCS cost shared on ‘conservation tillage’ through the Environmental Quality Incentive Program (EQIP) if the farmers could show a 40% reduction in tillage passes. It was unclear in the survey how farmers were interpreting the definition of CT and if pass reduction facilitated by the installation of a subsurface drip irrigation system put them into the CT category thereby qualifying them for EQIP cost sharing. In farmer interviews, one farmer described his tomato operation as CT because he was able to reduce passes by more than 40% while still using shallow tillage that did not preserve surface residues. This 40% reduction in passes practice is defined as CT by the California NRCS as well as the SJV Unified Air Pollution Control District that mandates farmers reduce dust.

### Why California farmers don’t use CT

Farmers consistently suggested that a barrier to adoption of CT is a lack of demonstrated economic justification. Previous work looking at adoption of sustainable agriculture practices in the Southern US also found that farmers most frequently cited economic reasons as the main barriers to adoption [62]. Another study reported that economics was generally cited as the main motivator guiding farm decisions, but when further interviews were done it became clear that farmer decision-making addressed many other considerations [63]. Our own published data on the economic benefits of CT systems in California indicate that potential cost savings of CT compared to conventional tillage range from $50 to $150 per acre depending on the crop [27, 57]. While such savings are by no means trivial or insignificant, they are often a relatively small part of overall production budgets for many California crops. However, because of the generally high intrinsic value of many SJV annual row crops, the risk of even a relatively small yield reduction,—for instance, two tons of processing tomatoes out of a typical 70 or more ton crop, would all but wipe out a potential savings of $150 per acre. Therefore, many CT non-adopters in California do not see cost-benefit tradeoffs of CT to be as yet favorable.

The technical and social barriers to CT reported in our study varied from farmer to farmer, but incompatibility with diverse vegetable crop rotations and demonstrated economic justification are two barriers that were definitively identified. Extensive development is needed to design diversified vegetable crop rotations that are compatible with CT practices in the SJV. Attitudes presented in Table 2 can be used to guide extension efforts in the process of encouraging innovation. Farmer ‘satisfaction with current practices’ points to the need for a longer-term motivation to transition to CT. Currently there is little perceived urgency to change practices, and so clarifying the longer-term economic return and potential to avoid future environmental regulations could help justify making such a significant transition. In addition and related to the economic benefits or cost savings that are likely to accrue with adoption of CT practices, economic studies on CT for a variety of SJV annual crops, suggest that production cost savings due to reductions in tillage passes, only account to $50 to $150 per acre per crop. These reductions in input costs may be relatively large with respect to crop budgets for agronomic crops such as corn, soybeans, and wheat, that have been the primary crops produced in regions where use of CT is more pervasive, however, they tend to be a far lower percentage of the production budgets for the more specialized rotation crops of the SJV.

### Why farmers adopt CT and the process of adoption used by current CT practitioners

Farmers who practiced CT did so because it was economically beneficial to them and improved their soil and cropping health. They described the process as requiring a shift in the mindset and commitment to learning. This mindset shift and a commitment to continuous learning in the CT adoption process described by farmers are important components of understanding CT adoption in California. Agricultural innovation has been defined as ‘a technological factor that changes the production function and regarding which there exists some uncertainty, whether perceived or objective (or both). The uncertainty diminishes over time through the acquisition of experience and information, and the production function itself may change as adopters become more efficient in the application of the technology. This definition facilitates the analysis of innovation diffusion as a dynamic process’ [64].

Farmer attitudes toward CT adoption in the SJV indicated large uncertainty. The above definition suggests that, on the innovation timeline, the uncertainty must be overcome with a high level of required ‘acquisition of experience and information,’ or learning, before widespread adoption of CT will be achieved. This is also consistent with Coughenour and Chamala (2007) [16] where they suggested that as CT is adopted, the practices themselves cannot be diffused because each farmer will have to make changes for their own situation. They further suggested that the process of on-farm innovation is what needs to be diffused.

**Fig 6:**
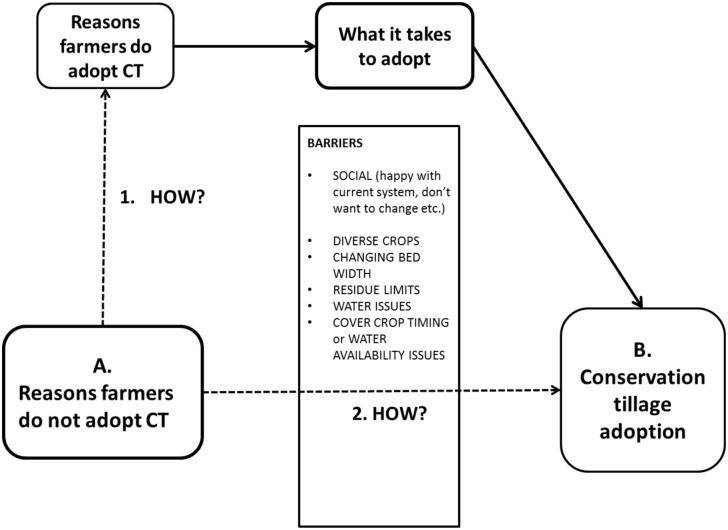
Flow chart of the two paths to CT adoption. (1) The path of farmers who have already successfully adopted CT. (2) The path of farmers that must address barriers particular to their farm or issues that have not yet been overcome by other farmers.

Fig 6 illustrates the current CT adoption scenario in California. Starting on the left with farmers who do not use CT, there are two paths to adoption. The first path is where farmers learn why other farmers use CT, try it and they are able to access enough information to successfully transition based on the experiences of other farmers. The second path is the more common route, where farmers are not adopting due to a number of barriers. In order to overcome these issues the farmer must go through the stage of ‘acquisition of experience and information,’ until they are able to successfully use the practice.

These scenarios imply that access and exchange of information will be essential for adoption. Perhaps more importantly they suggest that support for the learning process, adapting information to local challenges, and support for this experiential trial-and-error phase could greatly help farmers along the path to adoption. The process of running trials has a return in improved skill and potential monetary gain due to improved cropping practices [65] and farmer-generated location-specific information is highly valuable to farmers [59]. Farmers in the SJV reiterated the importance of locally-generated information and would like to see more local CT information. Adoption is dynamic and requires multiple pieces to come together including local, reliable information, demonstration, success by innovative farmers and learning-by-doing experiences that are within a community’s acceptable risk perception before it will take hold in a region [66].

## Summary and Conclusions

The diversity of crops and unique characteristics of California agriculture impacted many of the components of adoption and were different from the findings of the CT surveys conducted in other parts of the US. This study showed that in order to conduct effective research, outreach, and communication about CT, a universally acceptable definition needs to be developed. Within California, farmers, researchers, and agencies conducting outreach should clarify their definitions of CT. In addition to clarifying the local definition of CT, more local research is needed that contributes to understanding of the role of CT in SJV agricultural systems. The results suggested that, in the current context, larger and less diverse farms were more likely to use CT. Future research should address the reasons for lower adoption rates on smaller farms and work to create programs that support the interest of smaller farms to adopt CT. This study also demonstrated the need for evaluating CT extension activities that are being currently conducted in the SJV. Many farmers are not adopting CT is because of perceived risks involved or the incompatibility of CT practices with their current cropping system. To reduce this perceived risk, farmers and researchers must continue exchanging information and gaining meaningful experience to improve and develop these practices. Most of the surveyed SJV farmers expressed transition to CT as a continuous learning process. Interviews revealed the complexities of the innovation learning process and the significant finding that the current model of informing farmers and letting them figure things out may not be sufficient. More is needed because learning is more complex than a one-way, didactic interaction between extension agent and farmer. Extension education typically focuses on the first component of the learning process where information is exchanged from university research to farmers. Our research suggests that gaining meaningful experience with CT practices by researchers in the local context is also a large component of successful adoption.

## Supporting Information

S1 File CTWorkgroupSurvey2001PLOSONE. (DOC)

S2 File Interview Questions. (DOCX)
