# Supplementary material for: The Potential for Conservation Tillage Adoption in the San Joaquin Valley, California: A Qualitative Study of Farmer Perspectives and Opportunities for Extension
Source: PLoS One. 2016 Dec 1;11(12):e0167612. doi: 10.1371/journal.pone.0167612 (PMC5132313; doi:10.1371/journal.pone.0167612)
Supplement: S1 File — (DOC) [file pone.0167612.s001.doc]

**Conservation Tillage Practices Survey**

Winter 2011

Conservation tillage involves no-till, strip-till, or minimum tillage systems that reduce overall tillage passes by at least forty percent relative to your region’s conventional tillage practices.

1. Are you a farmer?  Yes  No

If you are not a farmer, what is your connection to farming? (equipment dealer, chemical sales, etc.)

2. Does your farm currently use conservation tillage?  Yes  No

3. Have you ever used conservation tillage?  Yes  No

4. How much do you know about Conservation Tillage? (1 = Nothing; 7 = A great deal)

1 2 3 4 5 6 7

5. Whether you use conservation tillage or not, please respond to the following statements based on what you “believe” about conservation tillage. Please rate these statements on a seven-point scale, where “1” is strongly disagree and “7” is strongly agree.

|  | Strongly Strongly  Disagree Agree | | | | | | | Don’t Know |
| --- | --- | --- | --- | --- | --- | --- | --- | --- |
|  |  |  |  |  |  |  |  |  |
| Conservation tillage is too risky. | 1 | 2 | 3 | 4 | 5 | 6 | 7 | DK |
| Converting to conservation tillage requires too much new equipment. | 1 | 2 | 3 | 4 | 5 | 6 | 7 | DK |
| Conservation tillage requires too many changes in what I’m currently doing. | 1 | 2 | 3 | 4 | 5 | 6 | 7 | DK |
| There is not enough demonstrated and successful experience with conservation tillage in California. | 1 | 2 | 3 | 4 | 5 | 6 | 7 | DK |
| Conservation tillage requires too much equipment “know how” and attention. | 1 | 2 | 3 | 4 | 5 | 6 | 7 | DK |
| There is not enough technical information and support available for conservation tillage in California. | 1 | 2 | 3 | 4 | 5 | 6 | 7 | DK |
| Conservation tillage will not work in California soils where there are no freeze-thaw conditions like in the Midwest. | 1 | 2 | 3 | 4 | 5 | 6 | 7 | DK |
|  | Strongly Strongly  Disagree Agree | | | | | | | Don’t Know |
|  |  |  |  |  |  |  |  |  |
| Conservation tillage is really not suited to California’s very diverse crops. | 1 | 2 | 3 | 4 | 5 | 6 | 7 | DK |
| The costs of converting to conservation tillage are too high. | 1 | 2 | 3 | 4 | 5 | 6 | 7 | DK |
| Conservation tillage fields will not yield as well as conventional tillage fields. | 1 | 2 | 3 | 4 | 5 | 6 | 7 | DK |
| I don’t know enough about conservation tillage equipment so that I can work on it if it breaks. | 1 | 2 | 3 | 4 | 5 | 6 | 7 | DK |
| I am satisfied with my current practices. | 1 | 2 | 3 | 4 | 5 | 6 | 7 | DK |
| Conservation tillage won’t work with my irrigation systems. | 1 | 2 | 3 | 4 | 5 | 6 | 7 | DK |
| Conservation tillage is being promoted by environmentalists. | 1 | 2 | 3 | 4 | 5 | 6 | 7 | DK |
| The “benefit/risk” case for conservation tillage in California has not been proven to me. | 1 | 2 | 3 | 4 | 5 | 6 | 7 | DK |
| Conservation tillage practices will likely result in unacceptable yield losses. | 1 | 2 | 3 | 4 | 5 | 6 | 7 | DK |
| My production practices are already about as “lean” as they can be. | 1 | 2 | 3 | 4 | 5 | 6 | 7 | DK |
| Conservation tillage results in more crop disease and weed-related losses. | 1 | 2 | 3 | 4 | 5 | 6 | 7 | DK |
| I don’t have the time to deal with learning about and securing new equipment for conservation tillage. | 1 | 2 | 3 | 4 | 5 | 6 | 7 | DK |
| Conservation tillage will not work for my crops. | 1 | 2 | 3 | 4 | 5 | 6 | 7 | DK |
| There is not enough public information available about conservation tillage in California. | 1 | 2 | 3 | 4 | 5 | 6 | 7 | DK |
| I really see little reason to change what I’m currently doing. | 1 | 2 | 3 | 4 | 5 | 6 | 7 | DK |
| Conservation tillage costs too much even with USDA cost sharing. | 1 | 2 | 3 | 4 | 5 | 6 | 7 | DK |

6. Please rate the following reasons on how important they were or would be in encouraging you to begin using conservation tillage. Please rate these statements on a seven-point scale, where “1” is “not at all important,” and “7” is “very important.”

|  | Not at all important | | |  | | | Very important | | | | Don’t Know |
| --- | --- | --- | --- | --- | --- | --- | --- | --- | --- | --- | --- |
|  |  |  |  | |  |  | |  |  |  | |
| Cut production costs | 1 | 2 | 3 | | 4 | 5 | | 6 | 7 | DK | |
| Reduce labor requirements | 1 | 2 | 3 | | 4 | 5 | | 6 | 7 | DK | |
| Use lower horsepower equipment | 1 | 2 | 3 | | 4 | 5 | | 6 | 7 | DK | |
| Have fewer tillage implements | 1 | 2 | 3 | | 4 | 5 | | 6 | 7 | DK | |
| Reduce dust emissions | 1 | 2 | 3 | | 4 | 5 | | 6 | 7 | DK | |
| Use less fuel | 1 | 2 | 3 | | 4 | 5 | | 6 | 7 | DK | |
| Add organic matter to the soil | 1 | 2 | 3 | | 4 | 5 | | 6 | 7 | DK | |
| Conserve water | 1 | 2 | 3 | | 4 | 5 | | 6 | 7 | DK | |
| Increase surface residues | 1 | 2 | 3 | | 4 | 5 | | 6 | 7 | DK | |
| Reduce greenhouse gas emissions | 1 | 2 | 3 | | 4 | 5 | | 6 | 7 | DK | |
| Reduce diesel emissions | 1 | 2 | 3 | | 4 | 5 | | 6 | 7 | DK | |
| Bring back earthworms to my fields | 1 | 2 | 3 | | 4 | 5 | | 6 | 7 | DK | |
| Take advantage of USDA cost sharing | 1 | 2 | 3 | | 4 | 5 | | 6 | 7 | DK | |

7. Please give your approximate age.

| 20 - 25 years old | 55 - 60 years old |
| --- | --- |
| 25 - 30 years old | 60 - 65 years old |
| 30 - 35 years old | 65 - 70 years old |
| 35 - 40 years old | 75 - 75 years old |
| 40 - 45 years old | 75 - 80 years old |
| 45 - 50 years old | 80 - 85 years old |
| 50 - 55 years old | 85 - 90 years old |

8. Roughly, how many years have you been farming?

| 0 - 5 years | 25 - 30 years |
| --- | --- |
| 5 - 10 years | 30 - 35 years |
| 10 - 15 years | 35 - 40 years |
| 15 - 20 years | 40 - 45 years |
| 20 - 25 years | 45 - 50 years |
| More than 50 years | |

9. What is the approximate size of your farm?

| Less than 100 acres | 1000 - 2000 acres |
| --- | --- |
| 100 - 500 acres | 2000 - 4000 acres |
| 500 - 1000 acres | 4000 - 10,000 acres |
| More than 10,000 acres | |

10. Which of the following crops do you grow?

| Alfalfa | Beans | Biofuel |
| --- | --- | --- |
| Corn | Cotton | Garlic |
| Lettuce | Onions | Sorghum sudan |
| Tomatoes | Triticale | Wheat |
| Others (please list) |  |  |

11. Do you use social network media such as ......

Facebook  Yes  No

You-Tube  Yes  No

MySpace  Yes  No

Linked-In  Yes  No

Twitter?  Yes  No

12. What is your approximate education level?

| High school |  |
| --- | --- |
| College / University BA or BS |  |
| Master’s degree |  |
| PhD degree |  |
| Other |  |

13. Which of the following are sources you routinely rely upon for agricultural information? Please rate your top five sources by indicating 1 = most important and 5 = lower importance.

| Local AM radio station |  |
| --- | --- |
| Internet / website |  |
| Email list serves or blogs |  |
| Private company media (pamphlets and brochures) |  |
| University extension service |  |
| NRCS Field Offices |  |
| Crop consultants |  |
| Public and university libraries |  |
| Farmer colleagues |  |

14. Please tell us in your own words why you do or do not use Conservation Tillage practices.

For additional information regarding the Conservation Tillage and Cropping Systems Workgroup, please see our Workgroup's website at <http://ucanr.org/sites/ct/> and contact Jeff Mitchell at (559) 303-9689 or [mitchell@uckac.edu](mailto:mitchell@uckac.edu).

We thank you for your time.
