# Supplementary material for: The Potential for Conservation Tillage Adoption in the San Joaquin Valley, California: A Qualitative Study of Farmer Perspectives and Opportunities for Extension
Source: PLoS One. 2016 Dec 1;11(12):e0167612. doi: 10.1371/journal.pone.0167612 (PMC5132313; doi:10.1371/journal.pone.0167612)
Supplement: S2 File — (DOCX) [file pone.0167612.s002.docx]

**Semi-Structured Interview Protocol**

For this interview, please answer each of the following interview questions as thoroughly and accurately as possible. We will use this information to better understand what the barriers are for adopting conservation tillage in California.

**Background Information**

1. Farming history –

- How did you start farming?
- When?
- Where?
- How long have you been farming on your current land?

2. Farm information

- How many acres?
- Ownership (own or lease)
- Which crops do you grow (and acreage)
  - Describe the management of each crop—tillage, irrigation, organic or conventional?
  - How much does this vary from year to year?
- Describe your soil
- Irrigation systems

1. How would you describe the type of farming that you do?
2. What is your approach to farm management?

- How do you think about your farming system (how are the crops you grow and the practices you use determined, what are the most important aspects of these decisions)
- How far in advance do you plan what you will grow?
- How often do you fallow a field?
  - Under what circumstance do you fallow?
  - How far in advance do you plan to fallow a field?
- Does the crop selection vary at all depending on the characteristics of the land (soil, water, ownership, lease)?
- Does the crop selection vary at all depending on contracts?
- How many passes do you do for particular crops?
  - How many of these are tillage operations?
- Which factors play the biggest part in deciding what to grow when and where?
- Do you have a written rotation plan for the farm?
- Do you keep written or computer records?
  - What do you include in these records?

5. How is your farm different from other farmers in your area?

6. What benefits do you feel farming provides?

**Part II. Decision Making Past, Present and Future**

Describe how you make decisions on your farm?

7. What previous decisions have had the biggest influence over the way your farm currently operates?

- Please describe when and why you made the decisions that you did?

1. How has each of these factors impacted how you make farming decisions in the past (positive or negatively)?

- Social (family, community, peers)
- Ecological
- Time (short term thinking vs. long term thinking)
- Industry

1. Which people have the biggest impact on the farming decisions that you make? (family, peers, organizations, industry)
2. Which decisions on your farm are unique to your operation and which do you feel are more general to the industry?
3. What do you think will be the biggest decision you will have to make in the near future of the farm?
4. How will each of these factors impact how you make farming decisions (positive or negatively) in the future?

- Social (family, community, peers)
- Ecological
- Time (short term thinking vs. long term thinking)
- Industry

**Part III. Information and Knowledge**

13. Generally, how do you get information about farming practices?

- Where (formats)
- What (topics)
- How frequently do you seek information?
- Who (people,organizations) ?

14. Which source of information do you rely on the most?

- Why?

15. What information could help you make better management decisions in the future?

16. Do you think the information exists, or would it need to be generated?

17. What sorts of record keeping systems do you have? (Written, electronic, mental)

- Who do share information with?
- Crop rotation, seed variety, cost and amt of inputs?
- Who you sell/buy from? And how much?

18. What sorts of experience do you have related to incentive programs?

- Which ones?
- Why?

19. Have you participated in any conservation programs (like EQIP)? Which ones?Have they been helpful?

- Why and why not? (How satisfied have you been with participation)
- Alternatives that would be more helpful?

20. Please describe in what ways you interact with other farmers?

- How many?
- How often?
- What type of information is exchanged?
- How often do you visit other farms?

**Part IV. Perceptions of Conservation Tillage**

**FOR FARMERS WHO DO NOT USE CT**

21. What do you know about conservation tillage practices?

- How did you find out about these practices? (Who? Where? When?)

22. Have you ever practiced or do you currently practice conservation tillage? Why or why not?

(probe common barriers- economic, equipment, knowledge, soil, residue, yields, happy with current practices, visual aesthetic)

*(ask 23 & 24 if YES to 22)*

1. If you have practiced any form of conservation tillage in the past can you please describe this experience? How much acreage? How long? Which crops? Describe techniques? Describe outcomes? Reasons for discontinuing?
2. When you were transitioning to CT what were the biggest challenges you faced?

- How did you try to overcome these challenges?
- What resources did you use?
- What resources could have made it easier to overcome these challenges?

1. Based on what you hear from other farmers what do you think non-adopters need to know more about in order to transition. Economics? HOW TO? Changes in timing and procedures? Finances? Number of years for transition to start consistently paying off?

26. What are the main reasons you choose not to use CT?

- Finances
- Knowledge (what do they need to know more about? Economics? HOW TO? Changes in timing and procedures? Finances? Number of years to start consistently paying off?
- no interest in changing / happy with current practices
- time
- soil
- residue management
- equipment
- “locked-in” through historical practices or industry

27. Which of these is the single biggest barrier for farmers who may want to adopt CT?

28. Can you identify any solutions to overcome these barriers to participation?

29. What sort of programs would have to happen to help make farmers transition to CT?

- Probe (incentives, policies, education, equipment sharing etc).

30. Where have you OR where would you go to get information about conservation tillage practices?

- Where (formats)
- What (topics)
- How frequently do you seek information?
- Who (people,organizations) ?

31. Which sources of information about conservation tillage do you trust the most?

- Why?

32. Is there important information about conservation tillage that isn’t available?

**FOR FARMERS WHO USE CT**

21. What is your experience with conservation tillage practices?

- How did you find out about these practices? (Who? Where? When?)

22. Describe your transition to conservation tillage practices? Why? How long? How many acres?

Challenges you faced? Reasons for sticking with it? Assistance?

1. Describe your current conservation tillage practice. How much acreage? How long? Which crops? Describe techniques? Describe outcomes? Reasons for continuing?
2. When you were transitioning to CT what were the biggest challenges you faced?

- How did you overcome these challenges?
- What resources did you use?
- What resources could have made it easier to overcome these challenges?

1. Based on what you hear from other farmers what do you think non-adopters need to know more about in order to transition. Economics? HOW TO? Changes in timing and procedures? Finances? Number of years for transition to start consistently paying off?
2. Think for a moment about farmers that do not use CT. What might be driving their unwillingness to participate?

- finances
- knowledge (what do they need to know more about? Economics? HOW TO? Changes in timing and procedures? Finances? Number of years to start consistently paying off?
- no interest in changing / happy with current practices
- time
- soil
- residue management
- equipment
- “locked-in” through historical practices or industry

27. Which of these is the single biggest barrier for farmers who may want to adopt CT?

28. Can you identify any solutions to overcome these barriers to participation?

29. What sort of programs would have to happen to help make farmers transition to CT?

- Probe (incentives, policies, education, equipment sharing etc).

30. As a farmer currently using CT would you be willing to mentor farmers who are transitioning to CT? (or do you already?)

- If yes- Under what circumstances?
- What could you offer to transitioning farmers?

31. Where have you gone and where do you go to get information about conservation tillage practices?

- Where (formats)
- What (topics)
- How frequently do you seek information?
- Who (people,organizations) ?

32. Which sources of information about conservation tillage do you trust the most?

- Why?

33. Is there important information about conservation tillage that isn’t available?
